# Breastfeeding Contributes to Physiological Immune Programming in the Newborn

**DOI:** 10.3389/fped.2021.744104

**Published:** 2021-10-21

**Authors:** Alberto Camacho-Morales, Mario Caba, Martín García-Juárez, Mario Daniel Caba-Flores, Rubí Viveros-Contreras, Carmen Martínez-Valenzuela

**Affiliations:** ^1^Departamento de Bioquímica, Facultad de Medicina, Universidad Autonoma de Nuevo León, San Nicolás de los Garza, Mexico; ^2^Unidad de Neurometabolismo, Centro de Investigación y Desarrollo en Ciencias de la Salud, Universidad Autonoma de Nuevo León, San Nicolás de los Garza, Mexico; ^3^Centro de Investigaciones Biomédicas, Universidad Veracruzana, Xalapa, Mexico; ^4^Unidad de Investigación en Ambiente y Salud, Universidad Autónoma de Occidente, Los Mochis, Mexico

**Keywords:** breastfeeding, maternal programming, microbiome, newborn, immunity

## Abstract

The first 1,000 days in the life of a human being are a vulnerable stage where early stimuli may program adverse health outcomes in future life. Proper maternal nutrition before and during pregnancy modulates the development of the fetus, a physiological process known as fetal programming. Defective programming promotes non-communicable chronic diseases in the newborn which might be prevented by postnatal interventions such as breastfeeding. Breast milk provides distinct bioactive molecules that contribute to immune maturation, organ development, and healthy microbial gut colonization, and also secures a proper immunological response that protects against infection and inflammation in the newborn. The gut microbiome provides the most critical immune microbial stimulation in the newborn in early life, allowing a well-trained immune system and efficient metabolic settings in healthy subjects. Conversely, negative fetal programming by exposing mothers to diets rich in fat and sugar has profound effects on breast milk composition and alters the immune profiles in the newborn. At this new stage, newborns become vulnerable to immune compromise, favoring susceptibility to defective microbial gut colonization and immune response. This review will focus on the importance of breastfeeding and its immunological biocomponents that allow physiological immune programming in the newborn. We will highlight the importance of immunological settings by breastfeeding, allowing proper microbial gut colonization in the newborn as a window of opportunity to secure effective immunological response.

## Introduction

In humans, the prenatal life (280 days) together with the following 2 years outside the womb (730 days) encompass “the first 1,000 days,” which define a physiologically plastic and vulnerable time-window where adverse health outcomes that may affect life in the future are programmed (1, 2). Women nutritional state before and during pregnancy have profound and long-lasting consequences for the proper development of the fetus, which is known as “fetal programming” (1). After birth, nutrition of an infant is critical to define the optimal growth, development, and future health of the individual later in life (1). Defective fetal programming promotes non-communicable chronic diseases in the newborn. Conversely, postnatal interventions such as breastfeeding during the first 1,000 days might mitigate risk factors and prevent metabolic and immune-related pathologies. In this regard, breast milk has been classified as the gold standard for infant nutrition during early postnatal life. According to the World Health Organization (WHO) and the United Nations Children's Fund (UNICEF), breastfeeding must provide nutritional support to the newborn no later than 1 h after birth and keep it as exclusive feed for at least the first 6 months, and then be supplemented with solid foods until 2 years of age or longer (2, 3). In fact, breastfeeding for the first 6 months of the life of an infant decreases the risk of overweight and obesity, type 2 diabetes (T2D), and other non-communicable chronic diseases in the infant (4–7).

Breast milk contains hundreds to thousands of distinct bioactive molecules that protect against infection and inflammation and contribute to immune maturation and proper organ development (8). Notably, breastfeeding also provides a source of bacterial colonization of the gut of the infant (9, 10). A healthy microbiota allows proper immune training in the newborn and immunogenic response under a future challenge in adulthood (9, 10). In contrast, using milk formula during lactation favors inadequate immune response and susceptibility to metabolic and immune-related pathologies in the newborn (1, 3). Also, maternal exposure to energy-dense foods might negatively change the immune composition of the milk and promote defective activation of the immunogenic response and immune maturation in the newborn (11–18). Overall, breastfeeding is a critical intervention that defines selective immunogenic programming settings and microbial colonization in the gut of the newborn, and prepares them to face several future health risks.

In the present contribution, we will focus on breast milk as the source of a plethora of bioactive molecules that provide immune maturation and healthy microbial gut colonization in the newborn. In a parallel scenario, we will provide compelling experimental evidence confirming that maternal exposure to energy-dense foods alter the immunogenic composition of breast milk and affect the microbiota in the newborn. We propose that immune acquisition through breast milk at early stages of life will provide functional microbial gut colonization preventing susceptibility to infection and negative outcomes of immunological activation.

## Prioritizing Breastfeeding For Healthy Newborn Maturation

Maternal breastfeeding has been practiced over millennia to secure good nutritional status for newborns (19). Breast milk is an extraordinarily complex, highly variable bioactive fluid, with changes in composition depending on the stage of lactation (from colostrum to late lactation), time of day, and physiological/nutritional state of the woman. Obstetric practices during labor play a critical role in initiation of effective breast feeding. For example, labor induced with oxytocin was negatively associated with effective breastfeeding initiation 36 h after birth, suggesting that induction of labor with oxytocin should be used judiciously (20). In fact, the World Health Organization (WHO) and the United Nations Children's Fund (UNICEF) recommend starting breastfeeding no later than 1 h after birth and that infants should be feed exclusively with breast milk for at least the first 6 months of life, and that breastfeeding should continue, supplemented with solid foods, until 2 years of age or longer (2, 3). Breastfeeding for the first 6 months of the life of an infant has enormous long-term health benefits, including prevention of the risk of metabolic-related comorbidities such as overweight and obesity, type 2 diabetes (T2D), and chronic diseases in the infant (4–7). Breastfeeding also improves positive metabolic outcomes in mothers (21–23).

Breast milk is a source of bioactive molecules, bacteria, and immune cells (8–10, 19). Immunogenic cells in breast milk program the immunogenic response in the newborn and incentivize healthy microbial colonization of the gut of the infant by training the immune system (8–10, 19). In this new scenario, breast milk protects the newborn against infection and inflammation at earlier stages of life and contributes to immune maturation (see below the section on *Breastfeeding contributes to physiological immunity in the newbor*). Notably, the role of breast milk in assisting physiological microbial colonization of the intestine of the infant occurs during the first 2 years of life (24), and there is evidence that altered gut microbiome in the newborn is found associated to metabolic compromise in children (25). Despite the many benefits of exclusive breastfeeding, only 40% of infants under 6 months are breast fed; only 23 countries have achieved exclusive breast feeding in at least 60% of infants <6 months old. Also, the Americas has one of the lowest breastfeeding rates worldwide, where only 6% of the countries have an exclusive breastfeeding rate above 60%. The rate of exclusive breast milk (EBM) in Mexico, according to the Encuesta Nacional de Salud y Nutrición (26), was 28% for infants under 6 months, one of the lowest in Latin America. We next add scientific evidence supporting the role of breastfeeding as a window opportunity to secure physiological immunity in the newborn preventing the risk to infection, immune tolerance, inflammatory immune profile, and microbiota disruption.

## Breastfeeding Contributes To Physiological Immunity In The Newborn

During the first weeks of postnatal life, the adaptative immune system of the newborn is immature, insufficient, and ineffective to protect against pathogens (27); multiple pathways have been proposed to explain defective immunity in the newborn, including immaturity of immune cells or lymphoid tissues. As a consequence, susceptibility to infections is elevated, and the probabilities of illness and death increase. In fact, birth is considered a dramatic and dangerous transition for the neonate, who is exposed to a new environment with a diverse microbial ecosystem compared with that *in utero*. Also, neonates experience enhanced susceptibility to infections while showing limited responsiveness to vaccination, particularly during the first months of life. Notably, the transfer of maternal immune components via breast milk allows the newborn to secure immunity to respond to any dangerous external pathogens, increasing their fitness for survival (28). Initial reports propose that women provide passive immune protection by transmitting antibodies in the colostrum during the first 2–4 days of breastfeeding (29, 30). Also, experimental evidence in human and murine models confirms several mechanisms involved in maternal immune transference to the newborn, such as maternal leukocyte transfer (MLT) (31), and microchimerism, the infiltration of maternal cells in newborn tissues (32).

**Figure 1:**
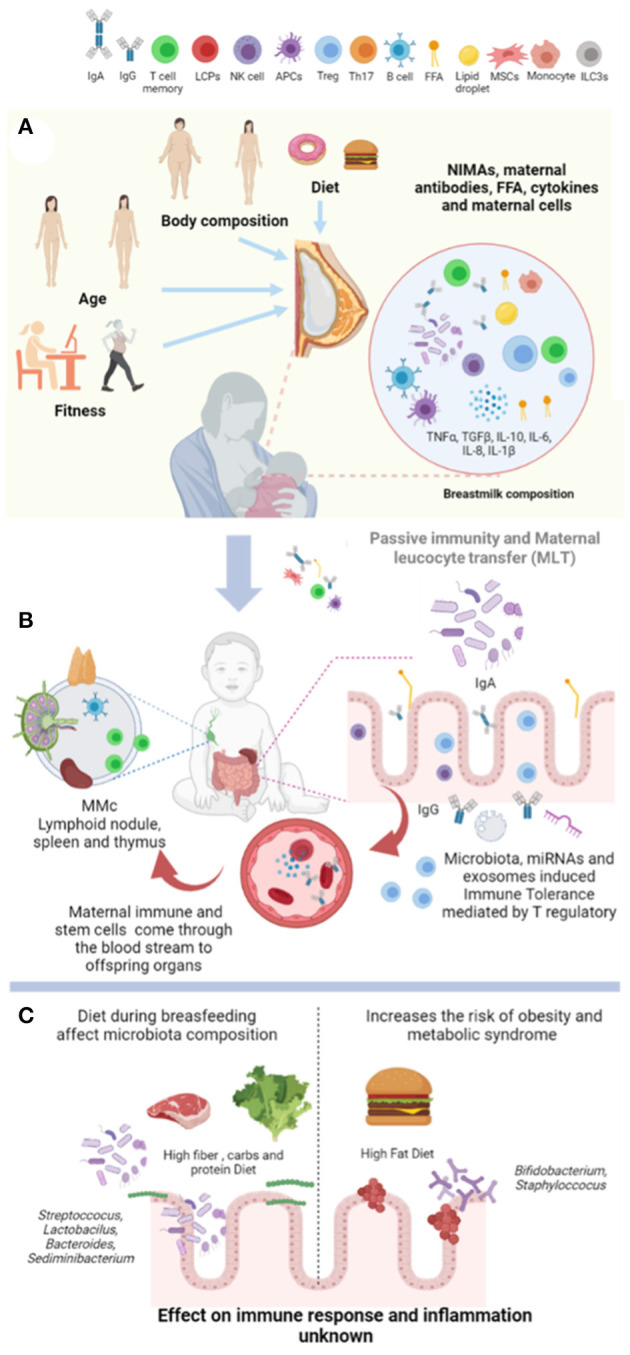
Breastfeeding provides immunological programming in the newborn. **(A)** Body weight, age, lifestyle, and diet quality influence breast milk composition such as lipid species, microbiota, cytokines, and accumulation of immune cell types. **(B)** Maternal antibodies, non-inherited maternal antigens (NIMAs), and maternal leucocyte travel through the stomach and intestine of the offspring. Also, maternal immune and stem cells invade the newborn blood leading to maternal microchimerism (MMc) to generate immune tolerance. Finally, microbiota, mRNAi, and exosomes provide immune tolerance by T-cell accumulation in the gut of the offspring. **(C)** High fat, carbs, and protein diets intake disrupts microbiota composition by promoting Staphylococcus and Bifidobacterium accumulation. Whereas, high fiber, carbs, and protein leads to lactobacillus microbiota. However, the effect of diet during breastfeeding on immune response, MMc, immune tolerance, and offspring microbiota establishment has not been fully determined in humans. NIMAs, non-inherited maternal antigens; MMc, maternal microchimerism. Created by Biorender.

Additionally, host mechanism such as self-missing, mediated by the natural killer cells of the newborn and the *de novo* production of neonatal immunoglobulin A (IgA) maintain intestinal microflora and immune adaptation (33, 34). Notably, breast milk immune composition seems to integrate a local secretion from multiple cell types, as well as peripheral production that not always correlate with blood levels (35). We propose that physiological routes that contribute to the newborn immunity are assisted by maternal breastfeeding (Figure 1).

## Breastfeeding Contributes To Proinflammatory Cytokine Profile In The Newborn

The components of breast milk and their role on proinflammatory profiles in the newborn have been described in recent years. Under homeostasis, proinflammatory cytokine profile in breast milk depends on gestational periods, maternal age, and maternal health (36). For instance, IL-6 and IL-8 were lower in breast milk at 36 weeks of gestational age (37), and TNF-α was observed only during the first few days of lactation (38). Some data report that colostrum in mothers with advanced age shows higher IL-1β and IL-6 levels when compared with adolescent mothers (36), confirming that aged mothers integrate a higher proinflammatory cytokine profile in their breast milk. There is also evidence that IL-6 accumulation in breast milk seems to depend on maternal IgA levels (29), suggesting that exposure to maternal infections or associated-cytokines might be accumulated in the breast milk to help the infant to survive. For instance, Type I-IFN accumulation in breast milk has been found after infection with influenza virus (39), whereas IL-10 and TGF-β decreased in mothers with allergies (40). In women with preeclampsia, high cytokine levels in breast milk persist for at least 30 days (41), and IL-1 and IL-6 increase, whereas IL-12 decreases in the colostrum (37). This evidence confirms that proinflammatory cytokine profile in breast milk is modulated by previous exposure to infections, allergies pathological traits, and aging (Figure 1).

**Figure 2:**
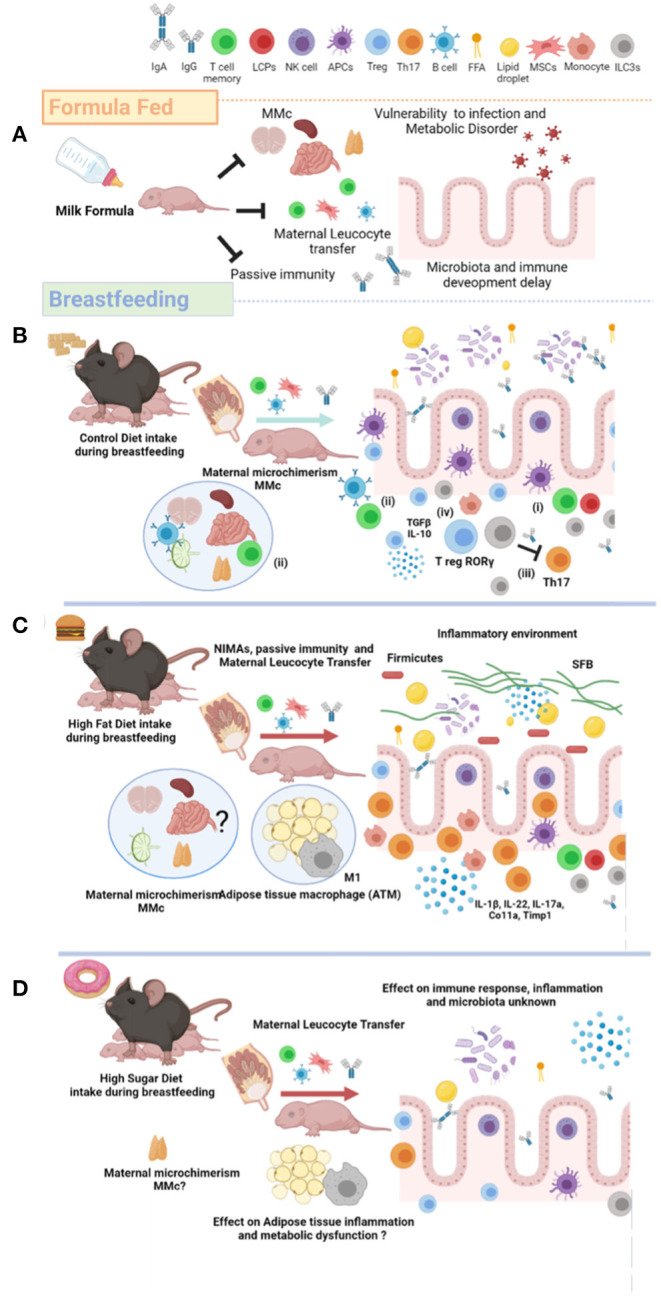
Maternal nutrition modulates breastfeeding composition and metabolic failure in the newborn. **(A)** Artificial milk feed formula does not promote innate and adaptative immune activation, maternal MMc, and gut microbiome development in the newborn. Defective immunological system leads to offspring vulnerability against viral and bacterial infection. **(B)** Breastfeeding from lean mothers or healthy maternal nutrition induce maternal antibodies (passive immunity), NIMAs, and leucocyte maternal transfer. Additionally, breastfeeding favors several cell mechanisms involved in immunogenic tolerance: (i) altered antigen presenting, (ii) specific T- and B-subtypes on MMc, (iii) Th17 cells suppression by ILC3s, and (iv) accumulation of T-regulatory cells and microbiota invasion. **(C)** HFD exposure during breastfeeding reduces ILC3s and Treg, and increases the TH17 I the gut. Breastfeeding of mothers exposed to HFD also increase the inflammatory cytokine profile, and SFB colonization and *firmicutes* in the gut of newborn. Also, HFD exposure during breastfeeding increases the M1/M2 macrophages ratio in adipose tissue (ATM). The effect of high fat diet (HFD) exposure during breastfeeding on the MMc has not been totally described. **(D)** The effect of high sugar diet on immunological programming in the newborn has not been totally described. MMc, Maternal Microchimerism; NIMAs, Non-Inherited Maternal Antigens; ILC3s, Type 3 Innate Lymphoid Cells; HFD, High Fat Diet; ATM, Adipose Tissue Macrophage; SFB, Segmented Filamentous Bacteria; FFA, Free Fatty Acid; HSD, High Sugar Diet; Treg, T-regulatory; Th17, T-helper 17; MSCs, Mesenchymal Stem Cells. Created by Biorender.

Preclinical analysis in murine models have also confirmed the effect of breastfeeding on the proinflammatory cytokine profile in the newborn. Precisely, a high concentration of TGFβ-1 has been detected in the milk of mice and in various tissues in the mouse pups (42), whereas the low concentration of cytokines such as IFNγ, IL-2, IL-4, IL-5, TNFα, and IL-13 were detected under healthy condition (33). According to these data, proinflammatory cytokines are present in breast milk, and they are essential for healthy development in newborns; during aging, however, a swift proinflammatory profile is exacerbated, which might provide adverse outcomes in the physiology of the newborn (Figure 2).

## Breastfeeding-Related Microchimerism Provides Immunogenic Cell Transfer To The Newborn

The transference of maternal cells such as immunogenic types, somatic tissue-specific cells, and stem cells to neonatal circulation and subsequent establishment in the newborn organs is known as maternal microchimerism (MMc) (43, 44). By itself, MMc establishes that the newborn displays a low frequency of immunogenic cells traveling and allocating in the tissues, but these cells might be retained for a long period of time (45). Notably, the MMc is involved in the tolerance, priming, and surveillance of the newborn, accounting as a major contributor of immunity after birth (46). Initial reports documented that maternal immune cells can be transferred to the fetus via the placenta during embryonic development (47). After birth, breastfeeding provides the newborn with immunogenic cell types that can remain until adulthood, as lymphoid and myeloid compartments of peripheral blood in healthy adult women (48, 49). Preclinical and clinical models documented that breast milk possesses several immunogenic cell types typically found in the blood, such as myeloid precursor cells, dendritic cells, and macrophages (33).

Additionally, innate lymphoid cells (ILCs) (34), natural killer (NK) (50), cytotoxic T cells (32), and T regulatory cells (51) have also been identified. Notably, recent evidence in humans shows that breast milk composition includes stem cells, specific memory CD4+ and CD8+ T-cells (52), and a large stem progenitor-like cell subset that expresses the CD45+ and CD45– markers (53). The CD45 marker is a transmembrane protein expressed in differentiated hematopoietic cells and seems to be an essential regulator for T- and B-cell antigen receptor signaling (53). On their own, murine models have confirmed findings in humans and demonstrated that breast milk from mice has B-cells with higher percentages of class-switched IgD-memory B-cells and plasma cells (PCs) (42, 43) and mammary gland IgA secretory cells (54), confirming immunogenic transmission to the newborn (Figure 1).

Although the mechanism involved in how immune cells travel from breast milk to the newborn circulatory system in humans is still not established, some potential pathways have been proposed. Evidence in humans suggests that breast milk components interact with the newborn saliva (31) protecting immune cells from the acidic pH of the stomach, and then, these cells infiltrate into the gut mucosa and travel to blood circulation within the newborn. Preclinical models have confirmed that the final allocation of maternal immune cells in mouse pups is an establishment of T-cell repository on the thymus, lymphatic nodes (55), spleen (56), Peyer's patches (32), brain (57), and gut (51).

Molecular and cellular regulation of MMc and elimination of non-inherited maternal antigens (NIMA) have been a matter of intense research. Preclinical murine models have provided important advances in the field of immunogenic transfer of maternal cell types to the newborn. Microchimerism and NIMAs were first reported in allogeneic transplantations. Reports propose several immune cell-dependent pathways of regulation: (i) antigen-presenting cells (APCs) NKs, B-specific phenotype and T-lymphocyte subset, and (ii) mesenchymal and stem cells. Molecularly, APCs from the newborn bind to the soluble antigen of maternal cells, allowing antigen processing and evasion of immune activation and systemic tolerance (44). Also, in a mice model of allogeneic hematopoietic stem cell transplantation (HSCT), breastfeeding generates Foxp3^+^ regulatory T-cells that suppress anti-maternal immunity and persist into adulthood (58).

Additionally, neonatal NK cells favor MMc by mediating the recognition and elimination of maternal antibodies IgA and IgGs (59). This process is known as missing-self recognition antibodies, which involves the Fc and CD16 proteins (59). In addition, host dendritic cells and plasmacytoid dendritic cells process membrane alloantigen acquisition (mAAQ+), favoring tolerance mediated by a decrease in allopeptides-MHC complex presentation and PD-L1 and CD86 expression (60). MMc is also regulated by infiltration of lymphocyte precursors cells (61), and by selecting neonatal subsets of Th1, Th2, and Th17 lymphocytes (62). Finally, clinical and experimental evidence in humans and mice show NIMA exposure during pregnancy and breastfeeding potentiates transplantation tolerance later in life (58). On this context, the high mobility group box 1 (HMGB1) protein levels in maternal circulation favors tolerance in the newborn against mesenchymal and stem cells (MSCs)-derived NIMA transferred via breast milk (63). HMGB1 is a non-histone nuclear protein secreted as a proinflammatory factor by activated macrophages and monocytes, and reported in certain autoimmune diseases such as systemic lupus erythematosus (64). HMGB1 has been also involved in the activation and mobilization of MSCs in adult circulation (65), inducing immune tolerance toward MSC-specific antigens in the newborn (Figure 2).

This evidence confirms a bidirectional immune crosstalk between maternal breastfeeding to the newborn and highlights the role of MMc on immune tolerance in the newborn.

## Maternal Antibodies Transferred By Breastfeeding Allow Immune Tolerance In The Newborn

As previously commented, maternal antibodies in colostrum maintain the newborn immune defense against pathogens during their first weeks of life. In humans, IgA antibodies are grouped into IgA1 and IgA2 subclasses, which display tissue-selective expression (66). The IgA1 is the main antibody in the respiratory tract, saliva, serum, and skin, whereas the IgA2 is localized in the intestine (66). Maternal antibodies are mainly composed of two types of immunoglobulins: (i) secretory IgA antibodies (SIgA), involved in protection mediated by microorganism neutralization and agglutination (67) and, (ii) four subtypes that are expressed in human and mice as antigen-specific IgG antibodies (IgG1, IgG2, IgG3, and IgG4) (68) which are induced by maternal immunization (69). Physiologically, the mammary gland secretes dimeric IgA antibodies that bind to the polymeric Ig-receptor (pIgR) on the basolateral membrane of the mammary gland epithelium, and both are internalized via endocytosis. IgA antibodies-pIgR dimers are released by the apical membrane as secretory IgA (sIgA) to the breast milk (70).

The maternal antibody IgG1 displays a half-life of about 48.4 days in the human newborn; however, they might be found in the serum of 4- to 6-month-old infants (71). In contrast, IgA antibodies are continuously supplied through the breast milk from the mother to the newborn (72). Experimental evidence in mice has confirmed that maternal antibodies have a half-life from 7 to 16 days postnatal (73) and they even could be found in serum until 14 weeks of age (74). However, reports show that maternal antibodies (IgG) decline and do not protect the newborn at later stages (72). Time-dependent, antibody-producing B-cells have been found in the neonatal gut, which reaches a peak after 30 days of postnatal life (31), however, the adaptive response is still immature and has not had enough time to acquire immunogenic memory.

Additionally, recent evidence described a selective IgGs known as maternal natural antibodies produced by exposure to pathogens or maternal immunization (75), which might interfere with the humoral immune response of the infant (70). High concentrations of vaccine-induced maternal antibodies in the infant blunt the immune response after a challenge (76). In fact, the newborn experiences an inhibition of antibody generation, showing lower antibody count, and affecting neonatal immunity for up to more than 1 year (72). Of note, defective immunological response in the newborn is not dependent on the type of vaccine applied in the mother but it seems to integrate a common pathway that involves a cross-link interaction between the B-cell receptor (BCR) and the Fcγ receptor (FcγRIIB), both expressed on the surface of B-cells (72). In this context, maternal IgG antibodies in the newborn bind to the FcγRIIB receptor, blocking the antibody production in response to the BCR-antigen interaction in the B-cells.

This evidence confirms that early newborn immunity depends on maternal antibodies for protection (Figure 1), but how long does this protection last?

## Maternal T-Regulatory Cell Transmission To The Newborn By Breastfeeding

Maternal transmission of T cells to the newborn is a topic of intense debate, and murine models have provided important advances on this field. Initial reports suggested that a microbe-induced population of receptor-related orphan receptor gamma t (RORγ+) Tregs is essential in controlling gut inflammation, and they are able to be preserved up to day 7 after birth (77). Ramanan et al. (78) demonstrated that RORγ+ Treg percentages varied between C57BL/6 and BALB/c mice. C57BL/6 have relatively high percentages of RORγ+ among Foxp3+ Tregs (40–60%) in comparison with BALB/c mice (20%) (78). Other studies also determined that Treg cells are transmitted by the mother to the newborn after birth, remain stable for life, and become resistant to many microbial or cellular perturbations (79). In fact, Tregs transmission in breast milk and the abundance of RORγ+ Tregs in the newborn secure bacterial clearance and delayed inflammation (79). Other studies show that breastfeeding may duplicate Tregs compared with neonates who received milk formula, and that it promotes tolerance against non-inherited maternal antigens (51). This evidence suggests that T-regulatory cells pass through breast milk, favoring immunity in the newborn and second generations.

Together, this evidence supports the notion that breastfeeding sets physiological immunity in the newborn by transferring proinflammatory cytokines, immunogenic cell subtypes, T cells, and maternal antibodies. Besides, immunity after birth is also closely regulated by microbiota in the newborn, confirming a mutually dependent interface of maternal breastfeeding and microbiota ecosystem.

## Breastfeeding–Microbiome Interplay Modulates Immunity In The Newborn

It is well-recognized that the gut microbiome integrates the most critical immune microbial stimulation in the newborn. The establishment of a healthy gut microbiome plays a crucial role in early life, leading to a well-trained immune system, and an efficient metabolism in healthy subjects (80). Early reports suggest that the gut microbiome of an infant would attain an adult-like composition by the age of three, but recent studies have suggested that a well-developed microbiome may take a longer time (81). Our microbiome is abundant in the gut, skin, hair, ears, vagina, and the respiratory and urinary tracts; however, the gut by itself supports more than 90% of the total microbiome. Initially, it was considered that the uterus was sterile, but now it has been demonstrated that the microbiome establishment begins during intrauterine life. In fact, the placenta, amniotic fluid, fetal membranes, and umbilical cord blood contain live microorganisms, suggesting that bacteria in these tissues do not necessarily indicate a pathogenic state but a symbiotic interplay (82). These data challenge the assumption of a sterile environment in the womb and indicate that initial colonization in the intestinal tracts of the infant can begin before birth. However, the gut microbiota of an infant is established mainly after birth in two transition periods in infancy: the first transition period occurs immediately upon birth, during breastfeeding, and results in dominance of the gut microbiome by *Bifidobacterium*, which is found in large quantities in breast milk (83). The second transition period occurs during weaning and establishes an adult-type complex microbiome dominated by the Phyla *Bacteroidetes* and *Firmicutes* (84–86). However, many other environmental factors, including cesarean delivery, medication, antibiotics, and maternal diet (including varieties of fibers), can alter the gut microbiome [(82); Figure 1].

Breast milk contains as many as 600 different bacterial species, up to 10^3^-10^4^ CFU/ml (87). It was proposed that bacteria may be transferred from mother to infant via breast milk through an “entero-mammary pathway” (88–94) to establish a healthy gut microbiome and populate the upper respiratory tract of the infant (88). The development of this respiratory tract microbiome, like that in the gut, is affected by the birth mode and the feeding practiced in childhood (88). Reports have documented that breast milk microbiome includes *Staphylococcus* and *Streptococcus*, as the most frequently cited taxa; however, additional taxa have been identified, including *Corynebacterium, Bifidobacterium, Propionibacterium, Bacteroides, Enterococcus, Faecalibacterium, Lactobacillus, Veillonella, Serratia, Ralstonia, Acinetobacter, Rothia*, and several members of the *Lachnospiraceae* and *Ruminococcaceae* families (95). Notably, *Staphylococcus, Lactobacillus, Enterococcus*, and *Bifidobacterium* found in breast milk microbiota are shared between mother-to-infant (10). In contrast, substituting breast milk with formula promotes a dramatic alteration of healthy gut microbiome establishment [(89, 90); Figure 1].

The microbiota in breast milk also modulates immunity in the newborn. Colonization of intestines by a diversity of bacteria in early life stimulates the differentiation and activation of T- and IgA-producing B cells that integrate the immune system in the newborn (91). Additionally, commensal microbiota is coated by IgA as a homeostatic IgA response, whereas humans express two subtypes (IgA1 and IgA2), mice express a single IgA subtype (92). Furthermore, maternal antibodies such as IgA transferred by breastfeeding stimulate the maturation of the innate mucosal immune system in the newborn in both humans and mice (80). IgA is a critical regulatory mechanism in this process of training and maturation of immunity. For instance, IgA-bacteria binding efficiently colonizes the small intestine (83), and according to Bunker and Bendelac (93), the bacteria are bound to a specific IgA, in the small, but not in the large intestine. By itself, maternal IgA in human has a relevant role on microbiome composition in the early months of life and is required for a healthy intestinal barrier and immune homeostasis. Also, maternal acquisition of antibodies in the newborn includes anti-commensal IgG2b and IgG3 by breast milk allowing activation of T-cell-independent and Toll-like receptor-dependent antibodies against their gut microbiota (59). This mechanism limits mucosal T-follicular helper response and germinal B-cell responses against new commensal antigens in the newborn (59). Also, maternal gut-associated lymphoid tissue (GALT) allows IgA accumulation stimulating the mammary gland to induce IgA secretion in the breast milk [(94); Figure 1].

Some studies in mice have identified selective microbiome species on acquired immunity in the newborn. Lactobacillus reuteri from the maternal microbiota is also found in breast milk and stimulates type 3 innate lymphoid cells (ILCs) in the lamina propria of the neonatal small intestinal to enhance IgA production (96). By itself, IgA plays a critical immunological role on the intestinal mucosa, in Peyer's patches, and in mesenteric lymph nodes of the newborn (97). Breastfeeding also favors the development of GALT by impairing mucosal immunity related to reduction of IgA levels through decreasing the IL-4 and IL-10 levels and the adhesion molecule MAdCAM-1 (98). CD4 T-regulatory cells (Tregs) subsets are essential to maintain self-tolerance in adult life as well in the neonatal period in humans and mice. Recently, reports documented that human breast milk promotes FoxP3^+^ Treg cell differentiation, increasing the number of FoxP3^+^CD4^+^CD25^+^ Tregs cell subtype and generating FoxP3 T-cell responses in the small intestine through microbiota (*Bifidobacterium breve, B. adolescentis, B. bifidum*, and *Lactobacillus plantarum*) (99). This evidence confirms a close relationship between secretory IgA and microbiota, allowing proper intestinal immune development in the newborn [(59); Figure 2].

Breast milk also contains hundreds of complex oligosaccharides and galactooligosaccharides, which contribute to the stability of the microbiome (100). Oligosaccharides in breast milk are more concentrated in the early stages of lactation, reaching up to 20–25 g/L in colostrum to 5–15 g/L in mature milk (101). Oligosaccharides-related molecules reach the colon of the infant, and are fermented mainly by *Bifidobacterium* to produce short-chain fatty acids (100). Oligosaccharides and probiotic components of breast milk modulate immune development, gut inflammation, and microbiome of the infants, conditioning their susceptibility to allergies (102).

Finally, altered early microbiome, called “dysbiosis,” might affect the development of the immune system of the host. Physiologically, the gut microbiome also maintains a constant crosstalk with the gut epithelia, inducing cell differentiation, and tight junction enhancement (103, 104); however, an imbalanced microbiome might destabilize the tight junction of epithelia, resulting in a leaky gut. The intestinal mucosal surface in the newborn shows differences with adult mucosa. For instance, the epithelium of the respiratory and gastrointestinal tracts of newborns has higher permeability (leaky) than those in adults, which increases the risk of tissue damage (80). A leaky epithelium allows an increased passage of toxic substances, bacteria, and viruses that might harm the body and increase susceptibility to pathological diseases. Besides, the epithelia of the newborn do not secrete enzymes or anti-microbial peptides, and the pH of the stomach and the composition and glycosylation of the secreted mucus layer also differ (105–107). By itself, dysbiosis increases the recruitment of immunological cell types, and activates the Toll-like receptors and nucleotide-binding oligomerization domain receptors, which exacerbate the release of inflammatory cytokines to the circulatory system (108–112).

Conversely, a healthy gut microbiome, from intrauterine life through the first 1,000 days, decreases the risk of suffering infectious and non-infectious diseases in early and late life. However, high-energy diets favor dysbiosis and negatively impact the gut microbiota (Figures 1, 2). We next add scientific evidence supporting the negative role of energy-dense diets or obesity in mothers on the immune programing of the newborn.

## Maternal Diet Modulates Breast Milk–Gut Microbiome Interplay And Immunity In The Newborn

Maintaining proper nutrition during lactation secures positive developmental and health outcomes in the newborn and in his adult life. Very few studies, however, have directly assessed the effect of maternal diets on immunogenic breast milk composition, and it remains as a very poorly understood topic. Also, the contribution of energy-dense diets on immune identity in breast milk and its effects on the microbiome of the newborn has not been completely explored. As Bravi et al. commented in their review, “the direct relation between the dietary intake of single nutrients and their presence within human milk has not been studied satisfactorily, for many reasons” (113). Some preclinical models have started to decode the impact of maternal diet on breast milk composition. Reports have documented that maternal diet during lactation modulates the composition of breast milk, glucose tolerance, and weight of the infant (7). Also, it had been confirmed that negative physiological conditions such as obesity, overweight, or overnutrition with energy-dense diets are associated with a pro-inflammatory profile and immune activation in the plasma of the infants after birth (114–118). In humans, 25% of calorie intake in obese people comes from snacks and junk food (119), which could have a negative contribution on breastfeeding composition and health in the newborns from obese mothers. Initial reports documented that supplementing the diet of lactating women with docosahexaenoic acid increases the concentration of docosahexaenoic and eicosapentaenoic acids in breast milk' however, there were no changes in IL-6 and TNF-α cytokines (11), or TGF-beta (12). In addition, supplementing the diet with black currant seed oil (BCSO) during pregnancy decreased IL-4 and increased IFN-γ levels in breast milk, whereas no significant differences were observed in IL-5, IL-10, IL-12, and TNF-α levels. Conversely, dietary intervention to increase consumption of fruits and vegetables during lactation in women decreases IFN-γ, TNF-α, IL-6, IL-8, and IL-1β (13).

Preclinical animal models have confirmed the deleterious effect of high-energy diets on pro-inflammatory cytokines accumulation in newborns. For instance, we and others have reported that maternal exposure to energy-dense diets in murine models increase peripheral pro-inflammatory cytokines such as TNF-alpha, IL-6, and IL-1β release and induce neuroinflammation in the newborn [(114, 118); Figures 1, 2]. Exposure to energy-dense diets programs maternal immune activation in mothers (120) and also shapes the microbiota in breast milk (15). As we commented, the breast milk microbiome harbors *Staphylococcus* and *Streptococcus* as the major families, as well as *Corynebacterium, Bifidobacterium, Propionibacterium, Bacteroides, Enterococcus, Faecalibacterium, Lactobacillus, Veillonella, Serratia, Ralstonia, Acinetobacter, Rothia*, and several members of the *Lachnospiraceae* and *Ruminococcaceae* families (95). Some reports suggest that maternal exposure to energy-dense diets modulates microbiota and immune profile in the breast milk, leading to major metabolic outcomes in the newborn (16). In addition, diets high in plant protein, fiber, and carbohydrates promote the presence of *Lactobacillus, Bacteroides, Sediminibacterium*, and *Streptococcus* in the microbiota of the breast milk, high intake of animal protein and HFD show accumulation of *Staphylococcus* and *Bifidobacterium* (15). Murine models also confirm that mothers exposed to HFD develop a selective microbiota profile by expanding firmicutes, a Gram-positive bacteria associated with promoting IL-17-producing type 3 innate lymphoid cells (ILC3s) in the lamina propia of the newborn, which seem to favor an increased susceptibility to intestinal injury (16). Notably, mice fed with energy-dense diets activate the aryl hydrocarbon receptor, a nuclear receptor/transcription factor involved in xenobiotic response that disrupts fat metabolism (17). Inhibition of ILC3s promotes intestinal inflammation mediated by increases in Th17 and IL-22 and colonization of segmented filamentous bacteria (SFB) (121). SFB are Gram-positive commensal, spore-forming bacteria found in mice and rat ileum, promoting the robust differentiation of T-helper-17 cells (Th17) (18). This suggest that diet components might also be recognized as xenobiotic elements and disrupt basal physiological settings, allowing intestinal inflammation. While these reports confirm that exposure to energy-dense diets modulates the immune response in the newborn by Th17/ILC3s-Treg balance in mice and microbiota profile in both, the effect of high sugar intake during breastfeeding is unknown (Figure 2). In an elegant recent report, Taylor et al. documented a new deleterious outcome associated to intestinal function in high sugar diets (122). The authors reported that exposure to dietary fructose improves the survival of intestinal cells, favoring the expansion of the surface area of the gut, and increasing nutrient absorption and adiposity in mice exposed to energy-dense diets.

Together, this evidence confirms that intake of energy-dense diets modulates the microbiome in the breast milk, allowing immune activation in the newborn.

## Breast Milk From Obese Mothers And Their Effects In Newborn Immunity

Breast milk from obese mothers displays a proinflammatory profile and contributes to neurodevelopmental alterations in the newborn. Reports confirm that maternal body mass index correlates with higher omega-6 to omega-3 fatty acid ratio and lower concentrations of lutein and docosahexaenoic, eicosapentaenoic, and docosapentaenoic acids in the breast milk (123). The authors confirm that concentrations of saturated fatty acids and monounsaturated fatty acids in breast milk were positively associated with maternal inflammation (123). In fact, breast milk from obese mothers is positively associated with a pro-inflammatory profile (123), suggesting that obesity contributes to breast milk composition and susceptibility to negative outcomes in the newborn.

Preclinical models have confirmed that breast milk from obese mothers partially protects the newborn against a challenge of high-fat diets (124). These data confirm that breast milk from obesity-prone dams fed with a high fat diet (HFD-OP) shows a decrease in the total lipid content and reduction in levels of the precursors of inflammatory lipids. Also, macrophage marker (F4/80), a marker of inflammation (TNF-α), a marker of tissue fibrosis, collagen-1 (Col1a), and tissue inhibitor of metalloproteinase-1 (Timp1) increase in the adipose deposits of 20-week old offspring mice exposed to HFD from obesity-resistant (OR) mothers (124). Conversely, newborn breastfed from mothers exposed to high fat diet and re-challenged to HFD in adulthood showed an increase in total CD11c+ proinflammatory and CD11c– anti-inflammatory markers in adipose tissue macrophages (ATMs), and M1:M2 proinflammatory ratio [(125); Figure 2]. These results show that HFD exposure during lactation promotes controversial results associated with inflammatory mechanism involved in newborn physiology and metabolism. For a deep understanding of the immunological properties in breast milk of obese mothers, see (126).

## Conclusion

Our proposal adds experimental evidence confirming the contribution of breastfeeding as a rationale to set immunity in the newborn during the first 1,000 days. We propose that breastfeeding secure physiological immunity in the newborn preventing the risk to infection, immune tolerance, inflammatory immune profile, and microbiota disruption. We propose that the physiological crosstalk of breastfeeding–microbiota assists proper immunological programming after birth, providing a mutual interface for healthy outcomes. However, conditions such as obesity or maternal exposure to energy-dense diets disrupts the physiological microbiome in the breast milk, favoring microbiota imbalance in the gut, and defective immunity in the newborn. Together, we conceive that breastfeeding supports an early priming stage of postnatal immune maturation and microbiome colonization, integrating a window of opportunity for preventive and interventional measures. Future studies are warranted to explore the long-term benefits of external factors assisting proper immune performance in the newborn to prevent obesogenic pathologies later in life.

## Author Contributions

MC, AC-M, MG-J, MDC-F, RV-C, and CM-V contributed to conception, design of the manuscript, and wrote sections of the paper. AC-M and MG-J design the figures. All authors contributed to the article and approved the submitted version.

## Funding

This study was funded by the COVEICYDET (151746) to MC.

## Conflict of Interest

The authors declare that the research was conducted in the absence of any commercial or financial relationships that could be construed as a potential conflict of interest.

## Publisher's Note

All claims expressed in this article are solely those of the authors and do not necessarily represent those of their affiliated organizations, or those of the publisher, the editors and the reviewers. Any product that may be evaluated in this article, or claim that may be made by its manufacturer, is not guaranteed or endorsed by the publisher.
